# Evaluating the reliability and information quality of ChatGPT responses on vaccine hesitancy: an expert panel study

**DOI:** 10.1186/s12889-026-28626-0

**Published:** 2026-07-21

**Authors:** Hande Can Güvercin, Cemal Koçak, Mehmet Furkan Aytekin

**Affiliations:** 1 Department of Public Health, Ankara University Faculty of Medicine, Ankara, Türkiye; 2 Division of Epidemiology, Department of Public Health, Ankara University Faculty of Medicine, Ankara, Türkiye

**Keywords:** Artificial intelligence, ChatGPT, Vaccine hesitancy, Health information quality, Expert evaluation, Large language models

## Abstract

**Background:**

Vaccine hesitancy is a major public health problem that threatens global immunisation programmes. The use of artificial intelligence-based chatbots to access health information is increasing. This study aimed to evaluate the responses of ChatGPT-4o to frequently asked questions about vaccine hesitancy in terms of health information quality, based on expert opinion.

**Methods:**

This cross-sectional expert evaluation study included 20 questions on vaccine hesitancy. These questions were submitted to ChatGPT-4o, and the responses were evaluated by nine experts from the fields of public health, paediatrics, and infectious diseases. The evaluation was based on six criteria synthesised from HONcode, DISCERN, JAMA Benchmarks, CRAAP, GQS, and QUEST: scientific accuracy, comprehensiveness, understandability, correction of misinformation, source attribution, and actionability. Each criterion was scored on a scale from 1 to 10. Inter-rater reliability was assessed using the intraclass correlation coefficient (ICC) and, as a prevalence-robust sensitivity analysis, Gwet’s AC2 with quadratic weights.

**Results:**

The highest mean scores were found for understandability (9.4 ± 1.1) and scientific accuracy (8.9 ± 1.3), while the lowest mean score was found for source attribution (4.5 ± 3.4). At the question level, the highest scores were obtained for Question 4, on aluminium in vaccines, and Question 14, on claims that pharmaceutical companies endanger children’s health by producing and promoting vaccines. The lowest scores were obtained for Question 5, on live vaccines during pregnancy, and Question 6, on natural immunity. ICC analysis showed moderate agreement only for source attribution (ICC = 0.582; *p* < 0.001); the low ICC values for the other criteria were attributable to a ceiling effect, as confirmed by Gwet’s AC2, which indicated substantially higher agreement for the high-scoring criteria.

**Conclusions:**

ChatGPT-4o can generate scientifically accurate and understandable responses to questions about vaccine hesitancy. However, it shows a systematic shortcoming in source attribution. Although the model has potential as a supportive tool in public health communication, its outputs should be reviewed by experts and supported with verifiable sources.

**Supplementary Information:**

The online version contains supplementary material available at https://doi.org/10.1186/s12889-026-28626-0.

## Background

Vaccine hesitancy is defined as a delay in acceptance or refusal of vaccination despite the availability of vaccination services [1]. It has become a global phenomenon that has attracted increasing attention, particularly after the COVID-19 pandemic [2]. Vaccine hesitancy may be driven by negative beliefs based on myths, exposure to misinformation, distrust in health systems or healthcare providers, the influence of opinion leaders, costs, geographical barriers, and concerns about vaccine safety. Addressing these fears and concerns is critical for increasing vaccine acceptance and reducing vaccine hesitancy [3].

Today, millions of individuals use the internet to obtain health information [4]. Studies have shown that artificial intelligence (AI) tools may serve as alternatives to search engines for accessing health information and, in some cases, provide more accurate results [5]. Research suggests that AI tools will play an increasingly important role in accessing health information and in other health-related areas [6]. As of July 2025, ChatGPT was the fifth most visited website globally and the most visited AI chatbot [7]. Furthermore, its growth rate and usage figures are markedly higher than those of other frequently visited platforms [8]. Although ChatGPT has several limitations, it has been reported as potentially beneficial for obtaining health information and delivering healthcare services [9]. The integration of AI tools such as ChatGPT into healthcare offers significant opportunities for improving public health [10]. However, uncertainties remain regarding the scientific accuracy and reliability of information generated by AI tools [11, 12]. Studies have demonstrated that health information obtained online can influence attitudes and behaviours towards vaccination [13]. Therefore, it is vital to address risks such as misinformation and content inconsistency while leveraging the benefits of AI [10].

This study aimed to evaluate the health information quality of responses provided by ChatGPT, an AI-based chatbot, to frequently asked questions about vaccine hesitancy under conditions designed to minimise personalisation and account-based biases, and to examine the role of such tools in public health communication.

## Methods

### Study design

This study was approved by the Human Research Ethics Committee of Ankara University Faculty of Medicine (Decision No: İ01-111-26). The study was designed as an expert panel evaluation to assess the responses of ChatGPT, an AI-based chatbot, to frequently asked questions about vaccine hesitancy. The questions used in the study were compiled from reliable sources, including the World Health Organization (WHO) [14], the United States Centers for Disease Control and Prevention (CDC) [15], Immunize.org [16], Historyofvaccines.org [17], and sources identified through a Google search using the keyword “vaccine hesitancy FAQ (Frequently Asked Questions).” Initially, each researcher independently compiled a list of frequently asked questions, resulting in a pool of 45 candidate questions. From this overlapping pool, three researchers rated each question as “relevant” or “not relevant” to vaccine hesitancy, with relevance judged by how directly a question reflected common concerns underlying vaccine hesitancy. Questions were retained by majority vote, and where several questions addressed a similar theme, the version considered most appropriate by the majority was selected. Through this process, 25 questions were excluded due to overlap or weak relevance, resulting in a final set of 20 questions (Supplementary Table 1). The questions were in English.

### Obtaining AI responses

Responses were obtained in English from the free version of ChatGPT-4o (GPT-4o, OpenAI) on 4 August 2025, from Ankara, Türkiye. Each question was entered verbatim as the sole prompt text in incognito browsing mode, without creating a user account and without any preceding instruction, system message, or framing. This approach aimed to reduce the influence of account history and personalisation on the responses. Each question was posed in a separate session, and the chat history was cleared before each query to prevent contextual carryover between responses.

### Data collection and management

The evaluation panel consisted of nine experts with 9–35 years of professional experience: two public health specialists, one public health specialist–epidemiologist, three paediatricians, and three infectious diseases and clinical microbiology specialists. All experts held academic positions ranging from assistant professor to professor, were native Turkish speakers based in Türkiye, and had advanced, academic-level English proficiency. The experts were identified through the researchers’ professional networks and were invited to participate. The experts evaluated the questions and responses independently, at different times and locations, and were blinded to one another’s scores. The questions, ChatGPT responses, and the scoring form were provided to each expert in printed format. No prior calibration or pilot scoring session was conducted; each expert applied the written criterion definitions and scoring instructions independently.

### Evaluation criteria

Responses were evaluated according to six criteria. These criteria were synthesised by reviewing the items of the Health On the Net Code of Conduct (HONcode) [18], the DISCERN instrument [19], the Journal of the American Medical Association (JAMA) Benchmarks [20], the Currency, Relevance, Authority, Accuracy, Purpose (CRAAP) test [21], the Global Quality Score (GQS) [22], and the Quality Evaluation Scoring Tool (QUEST) [23], which are widely used in the field of health information quality, and were tailored to the aims of this study. The criteria were as follows: Scientific Accuracy, Comprehensiveness, Understandability, Misinformation Correction, Source Attribution, and Actionability. Each criterion was scored on a scale from 1 (lowest) to 10 (highest). Before the evaluation, the experts were provided with a written form containing the definitions of the criteria and scoring instructions (Table 1).

**Table 1:** Evaluation criteria and definitions

Criterion	Definition
C1 – Scientific Accuracy	To what extent does the response align with current scientific evidence and accepted medical literature?
C2 – Comprehensiveness	Does the response address different aspects of the question with sufficient depth and clarity?
C3 – Understandability	Is the response written in a clear and plain language that is easily understandable to a general audience?
C4 – Misinformation Correction	Does the response clearly identify and correct any myths or misinformation if present?
C5 – Source Attribution	Does the response refer to reliable sources or organisations to support the information provided?
C6 – Actionability	Does the response offer the user practical and specific actions they can take based on the information?

### Statistical analysis

Statistical analysis was performed using R software (version 4.6.0). Descriptive statistics were reported as frequency, percentage, mean ± standard deviation, and median. Inter-rater reliability was assessed by calculating the intraclass correlation coefficient (ICC) using a two-way random effects model with absolute agreement for average measures [ICC(A, k)]. ICC values were interpreted according to the classification proposed by Koo and Li [24]: <0.50 poor, 0.50–0.75 moderate, 0.75–0.90 good, and > 0.90 excellent. As a prevalence-robust sensitivity analysis, Gwet’s AC2 coefficient with quadratic weights was calculated for each criterion alongside the ICC, given the known sensitivity of the ICC to restricted score variance. AC2 was computed using the irrCAC package in R. The level of significance was set at *p* < 0.05.

## Results

In this study, responses provided by ChatGPT to 20 frequently asked questions about vaccine hesitancy were evaluated by nine experts according to six criteria: C1, Scientific Accuracy; C2, Comprehensiveness; C3, Understandability; C4, Misinformation Correction; C5, Source Attribution; C6, Actionability. Each criterion was scored on a scale from 1 to 10.

**Fig. 1:**
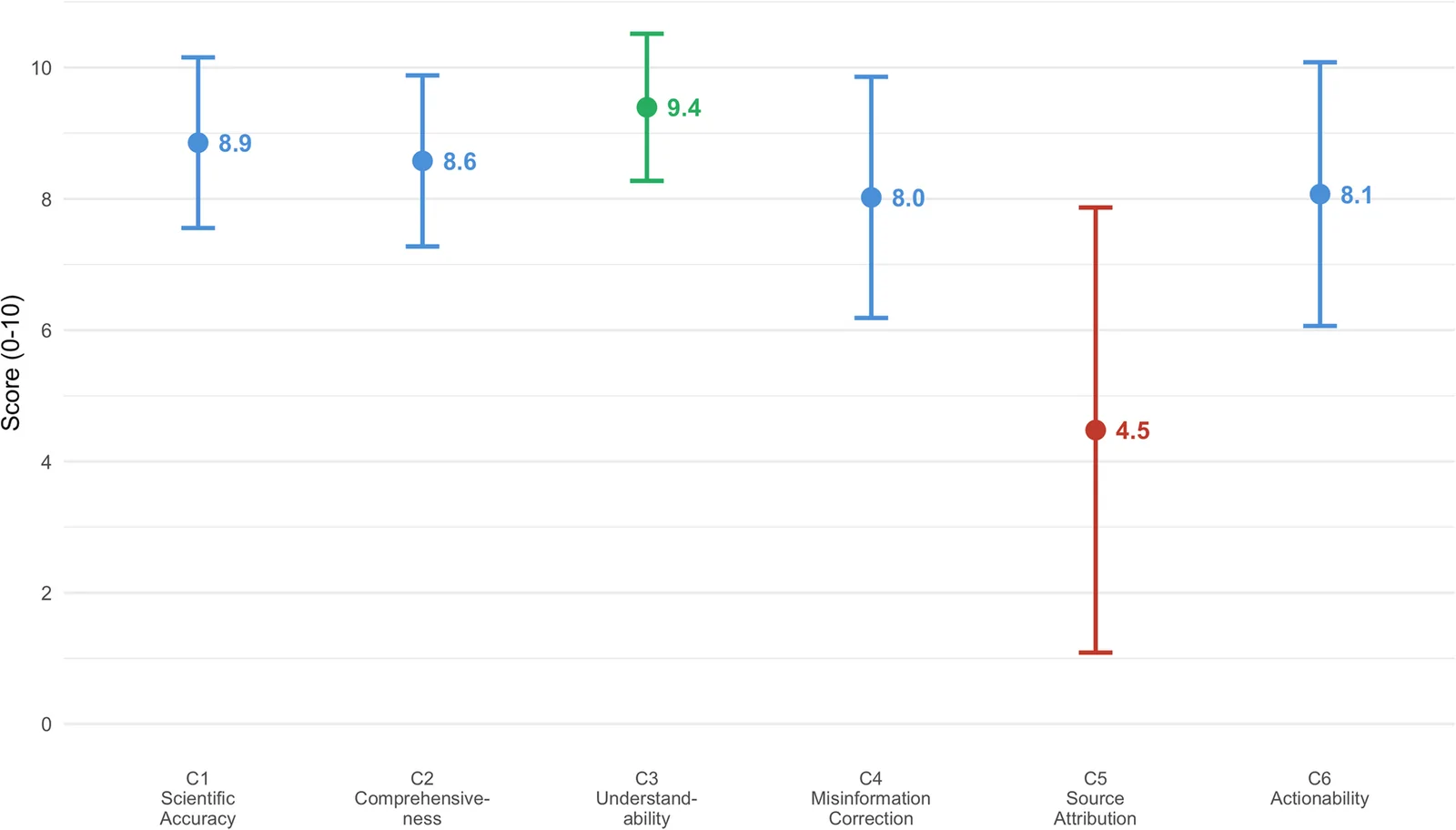
Mean scores and standard deviations (Mean ± SD) of ChatGPT responses by evaluation criteria. The highest-scoring criterion (C3, Understandability) is shown in green and the lowest-scoring criterion (C5, Source Attribution) in red; the remaining criteria are shown in blue

When all questions were evaluated together, the mean scores of ChatGPT’s responses were 8.9 ± 1.3 for Scientific Accuracy (C1), 8.6 ± 1.3 for Comprehensiveness (C2), 9.4 ± 1.1 for Understandability (C3), 8.0 ± 1.8 for Misinformation Correction (C4), 4.5 ± 3.4 for Source Attribution (C5), and 8.1 ± 2.0 for Actionability (C6). The highest mean score was observed for Understandability (C3) and the lowest for Source Attribution (C5). Mean scores and standard deviations by evaluation criteria are presented in Fig. 1.

The results of the inter-rater reliability analysis are presented in Table 2. The ICC(A, k) value for Source Attribution (C5) indicated moderate agreement (ICC = 0.582; *p* < 0.001), while Comprehensiveness (C2) and Scientific Accuracy (C1) showed poor agreement. For the remaining three criteria (C3, C4, and C6), ICC values were not statistically significant. The low ICC values observed for criteria with high mean scores and low variance, such as C1 and C3, may be explained by a ceiling effect. To quantify this, we calculated the combined proportion of scores of 9 and 10 for each criterion. This proportion was highest for Understandability (C3; 81.7%) and Scientific Accuracy (C1; 68.3%) - the two criteria with the lowest ICC values - and lowest for Source Attribution (C5; 19.4%), which showed the highest ICC. This inverse pattern between score concentration and inter-rater reliability supports the interpretation that the low ICCs for high-scoring criteria reflect restricted score variance rather than genuine disagreement among the experts.

As a sensitivity analysis, and because the ICC is sensitive to low score variance (the kappa paradox), Gwet’s AC2 with quadratic weights was additionally calculated for each criterion (Table 2). For the high-scoring criteria, AC2 yielded substantially higher agreement than the ICC (C3 = 0.702; C1 = 0.586; C2 = 0.554; C6 = 0.550), consistent with the interpretation that the low ICC values reflect restricted score variance rather than genuine disagreement among experts. For Source Attribution (C5), AC2 was close to zero (-0.005), indicating that - despite the moderate ICC driven by high score variance - experts did not consistently agree on absolute source-attribution scores across questions.

**Table 2:** Descriptive statistics and inter-rater reliability analysis results by evaluation criteria

Criterion	Mean ± SD	Median	ICC(A, k)	ICC 95% CI	ICC *p*	Gwet’s AC2*	AC2 95% CI
C1 – Scientific Accuracy	8.9 ± 1.3	9.0	0.272	0.04–0.55	**0.002**	0.586	0.48–0.69
C2 – Comprehensiveness	8.6 ± 1.3	9.0	0.352	0.06–0.64	**0.005**	0.554	0.45–0.66
C3 – Understandability	9.4 ± 1.1	10.0	0.006	–0.14–0.25	0.440	0.702	0.64–0.77
C4 – Misinformation Correction	8.0 ± 1.8	8.0	0.093	–0.08–0.36	0.145	0.280	0.17–0.39
C5 – Source Attribution	4.5 ± 3.4	4.0	**0.582**	0.29–0.80	**< 0.001**	-0.005	−0.24–0.23
C6 – Actionability	8.1 ± 2.0	9.0	0.107	–0.07–0.37	0.113	0.550	0.47–0.63

ICC(A, k): Two-way random effects model, absolute agreement, average measures. ICC interpretation: <0.50 poor; 0.50–0.75 moderate; 0.75–0.90 good; >0.90 excellent [24]. Bold values indicate statistically significant results (*p* < 0.05). All AC2 *p* < 0.001 except C5 (*p* = 0.52)

*ICC *Intraclass correlation coefficient, *AC2 *Gwet’s agreement coefficient (quadratic weights), *CI *Confidence interval, *SD *Standard deviation

**Fig. 2:**
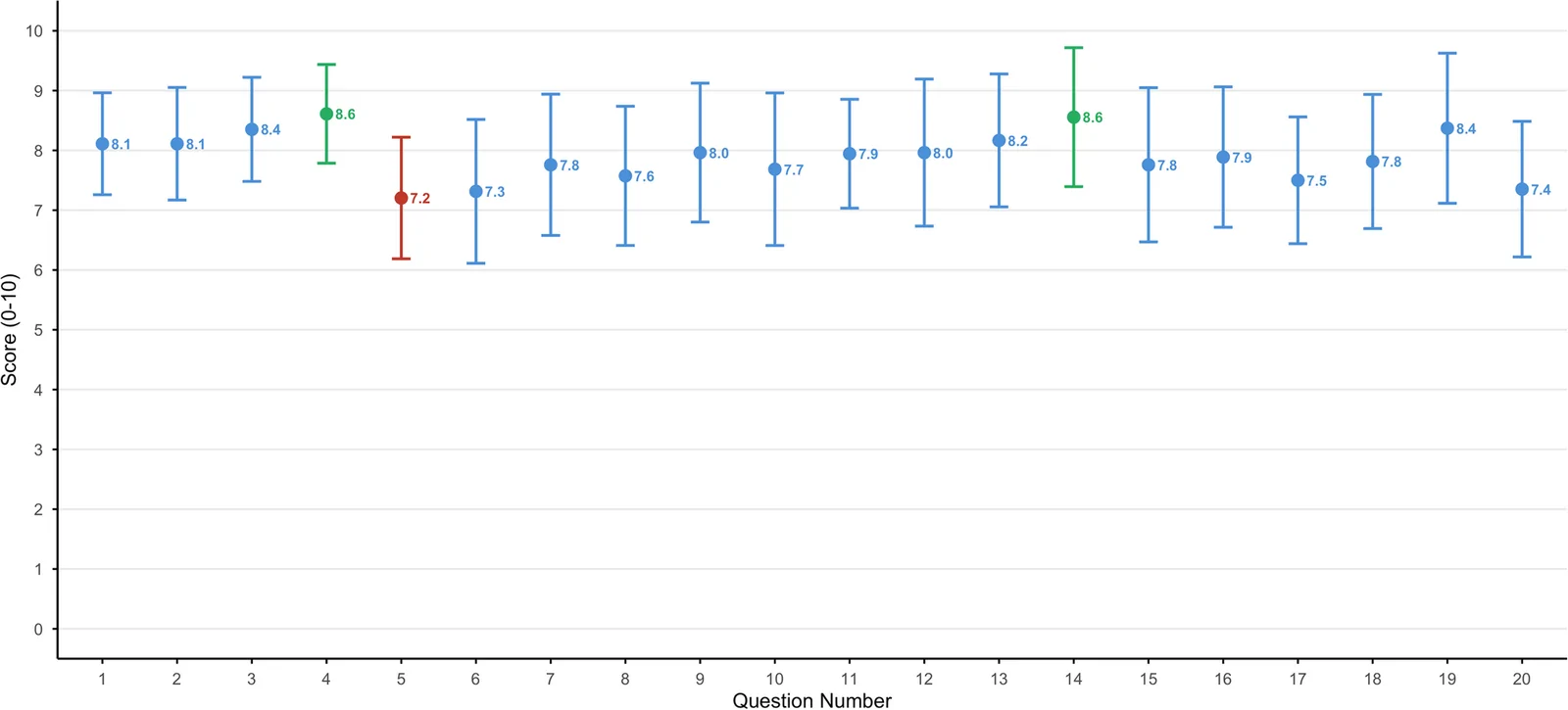
Question-level mean scores (averaged across all six criteria) with 95% confidence intervals. The highest-scoring questions (Q4 and Q14) are shown in green and the lowest-scoring question (Q5) in red; the remaining questions are shown in blue

Mean scores across all six evaluation criteria for each of the 20 questions are presented in Fig. 2. Question-level mean scores ranged from 7.2 to 8.6. The highest mean scores (8.6) were obtained for Question 4 (“Is aluminium in vaccines harmful?”) and Question 14 (“Is it true that big pharmaceutical companies knowingly endanger children’s health by producing and promoting vaccines?”), while the lowest mean score (7.2) was obtained for Question 5 (“Are live vaccines safe during pregnancy?”). Additionally, Question 6 (“Doesn’t acquiring natural immunity by getting the disease provide better protection than vaccination?”) and Question 20 (“Can I get sick from the viruses or bacteria in a vaccine?”), with mean scores of 7.3 and 7.4 respectively, were among the relatively lower-scoring questions. Given the limited panel size (*n* = 9) and the overlapping confidence intervals shown in Fig. 2, these question-level differences should be interpreted with caution rather than as definitive rankings.

**Fig. 3:**
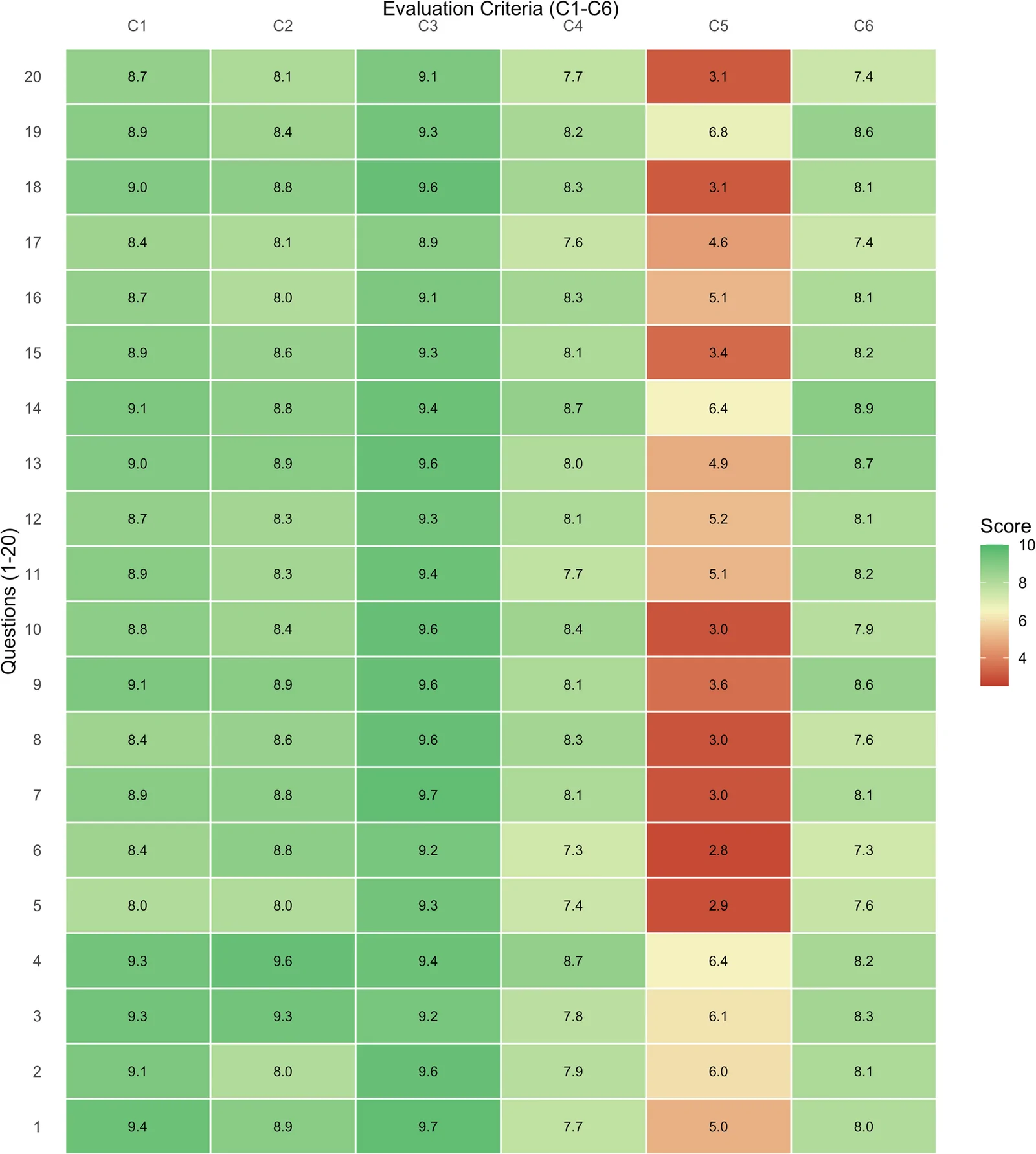
Heatmap showing mean scores of ChatGPT responses by question and evaluation criteria

The distribution of performance by question and criterion is presented in Fig. 3. The heatmap findings indicate that low scores for Source Attribution (C5) were not limited to specific questions but represented a systematic limitation observed across nearly all questions. In contrast, the consistently high scores for Scientific Accuracy (C1) and Understandability (C3) suggest that the model demonstrated strong and stable performance in these areas.

When the relatively lower-scoring questions (particularly Questions 5, 6, and 20) were examined, these questions were generally found to address more complex and context-sensitive topics such as pregnancy, live vaccine safety, and vaccine contents. For these questions, Source Attribution (C5) scores were notably lower (2.9, 2.8, and 3.1, respectively) compared to the other criteria. Conversely, for the highest-scoring questions (Q4 and Q14), Source Attribution (C5) was the criterion that differed most from its overall mean, rising to 6.4 in both questions compared with the criterion-wide average of 4.5. As the remaining criteria were consistently high across all questions, Source Attribution was the main criterion distinguishing both the highest- and lowest-scoring questions, further underscoring its central role in the overall variation in response quality.

A complementary analysis was conducted by recalculating question-level mean scores excluding C5. Even after this exclusion, Questions 5, 17, and 20 remained among the lowest-scoring questions, while Questions 4 and 14 remained the highest. Among the lowest-scoring questions, the criteria falling most below their overall means were Misinformation Correction (C4) and Actionability (C6), while Understandability (C3) remained high.

These findings indicate that ChatGPT’s responses to vaccine hesitancy-related questions demonstrated high performance in Scientific Accuracy and Understandability, while exhibiting notable limitations in Source Attribution.

## Discussion

In this study, responses provided by ChatGPT-4o to 20 frequently asked questions about vaccine hesitancy were evaluated by nine experts using six criteria. The results showed that the model performed well in Scientific Accuracy (8.9 ± 1.3) and Understandability (9.4 ± 1.1), while exhibiting a systematic shortcoming in Source Attribution (4.5 ± 3.4). These findings suggest that ChatGPT has the capacity to generate scientifically accurate and comprehensible content on vaccination, but has important limitations in supporting the information it produces with verifiable sources.

The performance of ChatGPT in terms of scientific accuracy on vaccination is consistent with similar studies in the literature. Torun et al. [25] compared the accuracy scores of ChatGPT’s responses to vaccine-related questions with those of physicians and found no statistically significant difference. Salas et al. [26] reported that 85.5% of the responses to 50 questions on COVID-19 vaccine safety were accurate. Fiore et al. [27] demonstrated that ChatGPT-4.0 and Gemini Advanced provided appropriate responses to 94.7% of 38 questions about vaccination myths identified by the WHO. These findings support the view that ChatGPT can produce scientifically reliable responses to vaccine-related questions. However, it should be noted that the model does not perform equally well in all health domains: Barut et al. [28] reported only 47.7% full concordance for rabies prophylaxis, while Bernal-Utrera et al. [29] found that 29% of responses regarding exercise recommendations for breast cancer were completely incorrect. On the other hand, Özcan et al. [30] found the responses on spinal cord injury to be generally reliable, although they identified deficiencies particularly in the general information section.

The most prominent finding of this study was the systematic shortcoming in Source Attribution (C5 = 4.5 ± 3.4). Heatmap analysis showed that this limitation was not restricted to specific questions but was consistently observed across nearly all 20 questions. This finding is directly related to one of the well-known limitations of large language models (LLMs), namely the “hallucination” problem: ChatGPT may cite non-existent articles as references or associate accurate information with incorrect sources. Similarly, Çitir [31] found that 10 of 81 references generated by ChatGPT-3.5 on oral cancer were fabricated. This limitation is particularly critical in the context of vaccine hesitancy, as gaining the trust of individuals with vaccine hesitancy requires the source of information to be transparent and verifiable. Therefore, the inability to provide reliable source attribution should be considered the most significant barrier to the use of ChatGPT as a standalone, trustworthy tool in public health communication.

The lowest-scoring questions, namely Q5 (live vaccines during pregnancy), Q6 (natural immunity), and Q20 (becoming ill from viruses or bacteria in vaccines), generally involved topics requiring clinical nuance and individualised risk assessment. This finding suggests that although ChatGPT performs well in presenting general medical information, its performance may decline in areas requiring individualised clinical judgement. Similarly, Koh et al. [32] reported that ChatGPT did not take into account individual sexual health factors or age-specific Advisory Committee on Immunization Practices (ACIP) recommendations, while Deiana et al. [33] noted that the model could misinterpret certain questions. These results reaffirm that AI cannot replace physicians’ clinical reasoning, and that patient–physician communication remains indispensable, particularly in situations requiring individualised risk–benefit assessment.

The inter-rater reliability analysis provided important findings for the methodological assessment of the study. Moderate agreement was observed only for Source Attribution (C5; ICC = 0.582), while ICC values for the remaining criteria were low. This can be attributed to a ceiling effect in criteria with high mean scores, such as C1 and C3: as the majority of experts assigned scores of 9–10 for these criteria, between-question variance remained extremely low, limiting the discriminative power of the ICC. Notably, the highest ICC value was observed for C5, indicating that experts consistently agreed on which questions performed better or worse in terms of source attribution. This serves as additional evidence supporting the main finding of the study, namely that source attribution represents a systematic weakness.

The relatively high score for Misinformation Correction (C4; 8.0 ± 1.8) is a practically important finding for addressing vaccine hesitancy. Misinformation is known to be one of the main drivers of vaccine hesitancy [34]. Sohail et al. [35] reported that ChatGPT provided responses supporting the scientific narrative on COVID-19 vaccines, while Singh [36] emphasised that although the model is promising, it is not sufficient on its own. In this context, the supervised use of AI tools such as ChatGPT by public health authorities - for example, their integration into official health organisations’ question-and-answer chatbots - could be an effective strategy for infodemic management. However, a meta-analysis by Wei et al. [37] reported an overall accuracy rate of 56% for ChatGPT’s responses to medical questions and identified high heterogeneity across studies (I² = 87%), indicating that the model’s performance can vary considerably depending on the domain and question type.

The performance for Misinformation Correction (C4) observed here should be interpreted in light of the recently documented tendency of large language models toward sycophancy - the inclination to affirm users’ stated beliefs rather than challenge them, even when those beliefs are inaccurate [38, 39]. This is particularly relevant to vaccine hesitancy: our questions were posed neutrally, but hesitant individuals typically frame queries as assertions of pre-held beliefs, and models have been shown to agree with incorrect user claims especially when these are expressed in assertive language [39]. Under such conditions, a sycophantic model may validate rather than correct a misconception. The moderate scores for Misinformation Correction (C4) observed here may therefore represent an upper bound, with real-world performance likely to be lower when users express pre-existing beliefs. Moreover, large language models have been shown to provide less accurate information to users with lower English proficiency and educational attainment [40], which raises equity concerns regarding their use in health communication.

Overall, the findings suggest that ChatGPT has potential as a supportive tool in public health communication on vaccine hesitancy; however, certain conditions must be met for this potential to be realised. These include expert review of model outputs, support from official health sources, and, in particular, stronger source attribution mechanisms. Several studies, including those by Biswas [41] and Ahn [42], have acknowledged the limitations of ChatGPT in the health domain and emphasised that its use should be complemented by other sources, particularly as providing accurate information alone is often insufficient to change vaccine-related attitudes [43]. For topics requiring clinical sensitivity, AI is best used as a clinician-mediated complement rather than a standalone information source. In practice, health professionals can play an active role by guiding patients toward authoritative sources, helping them interpret AI-generated information, and remaining the primary point of reference for clinically sensitive questions.

## Strengths and limitations

The questions used in this study were posed to the AI model in English, and responses were obtained in English. This may have contributed to higher performance because of the model’s extensive training data in English. The model’s performance in Turkish or other languages may differ owing to linguistic structures and local cultural variations, which represents a limitation. The generalisability of these results to non-English-speaking populations may therefore be limited. To address this, future studies could administer Turkish translations of the same prompts to a comparable expert panel, enabling a direct cross-language comparison of the health information quality of ChatGPT’s responses.

Large language models are continuously updated, and their algorithms change over time. The responses obtained in this study reflect the state of the model at a specific point in time. In future updates, the accuracy or tone of the model’s responses on vaccination may change, which could reduce the validity of these findings over time.

Another limitation of the study is the relatively small size of the expert panel (*n* = 9). Although the low ICC values for most criteria can be attributed to the clustering of scores within a narrow range, that is, a ceiling effect, the possibility that the evaluation criteria were not sufficiently discriminative should also be considered. Future studies should use larger expert panels and standardised evaluation tools supported by pilot testing. In addition, no calibration or pilot scoring session was conducted prior to the formal evaluation, which may have contributed to variability in how individual experts interpreted and applied the scoring criteria. Furthermore, as all experts were native Turkish speakers, their assessment of language-dependent criteria such as Understandability and Comprehensiveness may differ from that of a native English-speaking audience. Relatedly, the panel consisted exclusively of healthcare professionals; as understandability and comprehensiveness are ultimately experienced by the lay public, the absence of a layperson perspective may limit how well these criteria reflect the experience of the general population.

The model was not explicitly asked to provide sources when the questions were posed; therefore, the low source attribution scores reflect the model’s performance under a typical user scenario and may not represent its maximum source attribution capacity. Furthermore, although incognito mode prevented the use of account history and personalisation, it did not mask the geographic origin of the queries; ChatGPT could still infer the user’s location (Ankara, Türkiye) from the IP address. Responses may therefore have been influenced by location-based tailoring, and the use of a VPN or multiple geographic vantage points in future studies could help assess this potential effect. Relatedly, while this non-personalised, stateless setting was intentional, it may not capture how the model behaves for real users, as personalisation features such as ChatGPT’s memory function have been shown to substantially alter responses in practice [44].

One of the strengths of this study is that it is among the few studies evaluating ChatGPT’s responses on vaccine hesitancy using multiple criteria and an expert panel. Furthermore, the participation of evaluators from three different specialties - public health, paediatrics, and infectious diseases - enabled the findings to be examined from a multidimensional perspective.

## Conclusions

This study demonstrated that the health information quality of ChatGPT’s responses to frequently asked questions about vaccine hesitancy was high for five of the six criteria, while a systematic shortcoming was identified in source attribution. The model showed particularly strong performance in scientific accuracy, comprehensiveness, misinformation correction, and understandability, but its reliability was limited on topics requiring clinical nuance, such as pregnancy, live vaccines, and vaccine contents. These findings suggest that ChatGPT has potential as a supportive and informative tool in public health communication on vaccine hesitancy.

ChatGPT and similar AI tools may be used by public health institutions as supervised, supplementary communication tools, with particular value in the rapid correction of misinformation and the dissemination of comprehensible health messages. However, their outputs must be reviewed by experts and supported with official health sources. To this end, future models should incorporate approaches such as retrieval-augmented generation (RAG) [45], which enables the integration of verifiable sources. Further studies should also evaluate model performance across different languages and cultures, and mechanisms for referring users to health professionals should be strengthened for topics requiring clinical sensitivity. Rather than a sufficient solution on their own, these tools are best regarded as one component of a broader, professionally guided communication strategy.

## Supplementary Information

Supplementary Material 1.

## Data Availability

The datasets generated and analysed during the current study are available from the corresponding author upon reasonable request.

## Abbreviations

AI: Artificial intelligence
LLM: Large language model
ICC: Intraclass correlation coefficient
CI: Confidence interval
SD: Standard deviation
WHO: World Health Organization
CDC: Centers for Disease Control and Prevention
RAG: Retrieval-augmented generation
ACIP: Advisory Committee on Immunization Practices
