# Supplementary material for: Evaluating the reliability and information quality of ChatGPT responses on vaccine hesitancy: an expert panel study
Source: BMC Public Health. 2026 Jul 21;26:2986. doi: 10.1186/s12889-026-28626-0 (PMC13673675; doi:10.1186/s12889-026-28626-0)
Supplement: Supplementary file 1 [file 12889_2026_28626_MOESM1_ESM.docx]

**1-)Can a child receive multiple vaccines at the same time?**

ChatGPT:

Yes, a child can safely receive multiple vaccines at the same time. This practice is common and recommended by health organizations like the Centers for Disease Control and Prevention (CDC) and the World Health Organization (WHO).

Key Points:

Safety: Studies show that giving multiple vaccines at once does not overload a child’s immune system.

Effectiveness: Vaccines work just as well together as they do individually.

Convenience: Fewer doctor visits mean less stress for the child and caregivers, and it helps ensure the child stays on schedule with immunizations.

Immune System Capacity: A child’s immune system is capable of handling thousands of antigens (the parts of germs vaccines target) daily. The number in vaccines is tiny by comparison.

Common Examples:

At a typical well-child visit, a baby might receive:

DTaP (diphtheria, tetanus, pertussis)

IPV (polio)

Hib (Haemophilus influenzae type b)

HepB (hepatitis B)

PCV (pneumococcal conjugate)

Rotavirus (oral vaccine)

These are often given at the 2-, 4-, and 6-month appointments.

If you have specific concerns or your child has a medical condition, it's a good idea to discuss the vaccine schedule with their healthcare provider.

**2-)Do vaccines cause autism?**

ChatGPT:

No, vaccines do not cause autism.

This concern originally came from a 1998 study by Andrew Wakefield, which claimed a link between the MMR (measles, mumps, and rubella) vaccine and autism. However, that study was found to be deeply flawed, based on fraudulent data, and has been retracted by the journal that published it. Wakefield also lost his medical license as a result.

Since then, many large, high-quality studies involving hundreds of thousands of children around the world have found no connection between vaccines and autism.

In short:

Vaccines are safe and effective.

Autism has complex genetic and environmental causes, but vaccines are not one of them.

Let me know if you want to look at some of those studies or see expert statements from organizations like the CDC or WHO.

**3-) Is thiomersal in vaccines harmful?**

ChatGPT:

Thiomersal (also known as thimerosal) is a mercury-containing compound that has been used as a preservative in some vaccines and other medical products since the 1930s. The short answer is:

No, thiomersal in vaccines is not harmful in the small amounts used. Here's why:

🔬 What Is Thiomersal?

It contains ethylmercury, not methylmercury (the type found in some fish and known to be harmful in high amounts).

Ethylmercury is processed and eliminated quickly from the body, unlike methylmercury, which can accumulate.

🧪 What Do Scientific Studies Say?

Extensive research by organizations like the CDC, WHO, and Institute of Medicine (US) has found no credible evidence linking thiomersal in vaccines to serious harm, including autism.

The FDA and European Medicines Agency have reviewed the safety data and concluded that thiomersal-containing vaccines are safe.

🧠 Why Was It Removed from Most Childhood Vaccines (in the U.S. and other countries)?

As a precautionary measure in the late 1990s and early 2000s, it was reduced or removed from most childhood vaccines, not because it was proven harmful but to reduce total mercury exposure in infants.

Some multi-dose vials of flu vaccines may still contain thiomersal to prevent contamination.

💉 Are Vaccines Without Thiomersal Available?

Yes, many countries now use single-dose vials or formulations without preservatives.

If you're concerned, you can request thiomersal-free versions (especially for flu vaccines).

✅ Summary

Claim	Reality

Thiomersal causes autism False	– Multiple large-scale studies show no link

Thiomersal is toxic in vaccines False – Used in small, safe amounts as a preservative

All vaccines contain thiomersal False – Most childhood vaccines are now thiomersal-free

If you'd like, I can point you to the most recent safety reviews or help you find a vaccine formulation that avoids thiomersal.

**4-)Is aluminium in vaccines harmful?**

ChatGPT:

Aluminum is used in some vaccines as an adjuvant, which helps to enhance the body’s immune response to the vaccine. The amount of aluminum in vaccines is very small, and research indicates that it is safe at these levels.

Here are a few key points about aluminum in vaccines:

Aluminum as an Adjuvant: Aluminum salts, such as aluminum hydroxide, aluminum phosphate, and potassium aluminum sulfate, are commonly used in vaccines to improve their effectiveness. These substances help to "boost" the immune system's response to the antigen (the part of the virus or bacteria that triggers an immune reaction).

Low Amounts: The amount of aluminum in vaccines is very low compared to the amount people are exposed to from everyday sources like food, water, air, and even some medications. For example, the amount of aluminum in a single dose of a vaccine is typically much lower than what a person might ingest through their diet.

Safety: Extensive research, including studies by organizations such as the World Health Organization (WHO), the Centers for Disease Control and Prevention (CDC), and the U.S. Food and Drug Administration (FDA), has shown that the small amounts of aluminum used in vaccines do not pose a significant risk to health. The body is capable of safely processing and excreting aluminum, primarily through the kidneys.

No Evidence of Harm: There is no credible scientific evidence that the small amount of aluminum in vaccines is linked to long-term health problems. Studies have shown that aluminum exposure through vaccines does not cause neurotoxicity, autoimmune disorders, or other harmful effects.

Exposure Comparison: The amount of aluminum in vaccines is far less than the amount an infant or child might get from breast milk, infant formula, or even exposure to environmental sources like cooking utensils or cosmetics.

Concerns and Misconceptions

Aluminum and Autism: One of the more persistent misconceptions is that aluminum in vaccines could be linked to autism. This claim has been thoroughly studied, and no credible evidence has been found to support it. The original study that suggested this link was retracted due to serious methodological flaws and conflicts of interest.

Aluminum and Neurotoxicity: Concerns about aluminum causing neurological damage have been examined in various studies, but no evidence has been found that the small amount used in vaccines causes harm. The amount of aluminum in vaccines is far below levels known to cause neurotoxicity in laboratory studies.

In summary, the use of aluminum in vaccines is considered safe based on current scientific evidence. If you have concerns about vaccines or any specific medical questions, it’s always best to consult with a healthcare provider who can provide personalized guidance.

**5-)Are live vaccines safe during pregnancy?**

ChatGPT:

Live vaccines are generally not recommended during pregnancy due to potential risks to the developing fetus. These vaccines contain weakened forms of the virus or bacteria, which can potentially cause harm to a pregnant woman or her baby.

For example:

Live vaccines to avoid during pregnancy:

MMR (measles, mumps, rubella)

Varicella (chickenpox)

Yellow fever

Live attenuated influenza vaccine (nasal spray)

If a pregnant woman inadvertently receives a live vaccine, it's typically recommended to wait at least one month before trying to conceive. However, in many cases, the risk is considered low, and healthcare providers may monitor the situation closely.

Exceptions:

In some instances, a healthcare provider may decide that the benefits of a live vaccine outweigh the risks (for example, if a woman is traveling to an area with a high risk of disease). However, this would generally be rare and carefully evaluated on a case-by-case basis.

It's always best to consult with a healthcare provider before making any decisions regarding vaccination during pregnancy. They can provide guidance based on the individual's health status, risk factors, and the specific vaccine in question.

**6-)Doesn’t acquiring natural immunity by getting the disease provide better protection than vaccination?**

ChatGPT:

That's a common question, and it's important to look at it carefully and honestly. Here's a clear breakdown of how natural immunity and vaccine-acquired immunity compare:

Natural Immunity (from infection):

Yes, it can provide strong immunity — often specific to the variant or strain that caused the infection.

It may last a long time, though protection can wane, and reinfection is still possible, especially with viruses that mutate (like COVID-19).

However, getting the disease can be risky:

You may develop serious complications (e.g., pneumonia, organ damage, long COVID, or death).

You can spread the disease to others while infectious.

For diseases like measles, chickenpox, or COVID-19, the cost of gaining natural immunity may be very high in terms of health risk.

Vaccine-Acquired Immunity:

Vaccines stimulate your immune system to develop protection without causing the disease itself.

They're designed to target key parts of a virus or bacteria — sometimes more broadly than a natural infection does.

Immunity from vaccination may decline over time, but boosters can help, and it's generally safer than getting infected.

For some diseases (like tetanus), natural infection doesn’t provide lasting immunity, but vaccination does.

So, is natural immunity better?

It depends on how you're defining "better."

Stronger immune response? Sometimes — yes, natural infection can lead to broader immunity.

Safer route to immunity? Almost always vaccination — because it gives protection without the risks of severe illness or complications.

Best approach in practice:

For many diseases, the strongest and safest protection often comes from a combination of vaccination and natural immunity (so-called hybrid immunity), especially for respiratory viruses like COVID-19.

If you’re asking this in the context of a specific disease (like COVID-19, chickenpox, or measles), I can tailor the answer more precisely.

**7-)If hygiene, nutrition, and clean water can keep us healthy, do we still need vaccines?**

ChatGPT:

Yes, we still need vaccines, even if we have excellent hygiene, nutrition, and access to clean water. Here's why:

🛡️ 1. Hygiene and nutrition reduce risk — but don’t eliminate disease

Good hygiene and nutrition can help your immune system function well and lower the chance of getting sick. Clean water prevents many waterborne diseases. However, they cannot fully protect you from infectious diseases — especially those that spread person-to-person through the air (like measles or COVID-19).

🧬 2. Some diseases are extremely contagious

Diseases like measles, polio, and influenza spread so easily that even one infected person can start a large outbreak — even in clean, well-nourished communities.

Measles, for example, is one of the most contagious viruses known. Without vaccination, 90% of people exposed to it will get infected.

🦠 3. Vaccines work in ways hygiene can’t

Vaccines train your immune system to recognize and destroy specific pathogens before they make you seriously ill. Hygiene doesn’t do that — it only lowers exposure.

🌍 4. We’ve seen what happens without vaccines

In countries or communities that reduce or stop vaccinations:

Diseases return — even if hygiene is excellent.

Example: Measles outbreaks in the U.S. and Europe have occurred in well-developed areas with high standards of hygiene — but low vaccination rates.

✅ Summary:

Factor	Helps prevent illness?	Eliminates disease?

Hygiene	✔️ Yes	❌ No

Nutrition	✔️ Yes (supports immunity)	❌ No

Clean Water	✔️ Yes (for some diseases)	❌ No

Vaccines	✔️✔️ Yes (direct immunity)	✔️ In some cases (like smallpox)

🧪 Bottom Line:

Vaccines are essential. They don’t replace hygiene or nutrition — they complement them. All are needed for a healthy population.

Let me know if you'd like specific examples of diseases or data.

**8-)Do vaccines have any harmful effects?**

ChatGPT:

Vaccines, like any medical intervention, can have side effects—but for the vast majority of people, these are mild and temporary. Here's a breakdown of the potential effects:

✅ Common and Mild Side Effects

These are signs your immune system is responding as expected:

Pain, redness, or swelling at the injection site

Mild fever

Fatigue or headache

Muscle aches

These typically resolve within a few days.

⚠️ Less Common but Possible Side Effects

Allergic reactions: Rare, but can be serious (e.g. anaphylaxis). This is why people are monitored briefly after vaccination.

Febrile seizures (in children): Rare and generally not harmful long-term.

Guillain-Barré Syndrome (GBS): Very rare and has been associated with a few vaccines (like the flu vaccine), but the risk from the infection itself is often higher.

❌ Myths and Misconceptions

Some claims about vaccine harm are unsupported by scientific evidence:

Autism: Extensive research has found no link between vaccines and autism.

"Overloading" the immune system: The immune system handles far more challenges daily than any vaccine presents.

📊 Risk vs. Benefit

The risk of serious harm from vaccines is extremely low, especially compared to the diseases they prevent. For example:

Measles can cause brain swelling or death.

HPV can lead to cervical and other cancers.

COVID-19 can cause long-term complications or death.

🔍 Summary

Vaccines are safe and highly regulated. While they can cause side effects, serious harm is rare, and the benefits overwhelmingly outweigh the risks for nearly all populations.

Would you like information on a specific vaccine or concern?

**9-)Why are so many vaccines given to babies? Isn’t it too much for their immune systems?**

ChatGPT:

That’s a thoughtful question, and it’s one a lot of parents and caregivers ask.

Why so many vaccines are given in infancy:

Babies are vulnerable: Newborns and young infants have immature immune systems. They’re more susceptible to serious complications from infectious diseases like whooping cough, measles, and meningitis. Vaccines help protect them during this vulnerable window.

Timing is strategic: Vaccines are scheduled based on when a child is most at risk and when the vaccine will be most effective. Some diseases are especially dangerous in infancy, so delaying vaccines increases the risk of harm.

Long-term protection: Early vaccination doesn’t just protect infants in the short term — it also primes their immune system for stronger, longer-lasting immunity as they grow.

Is it too much for their immune systems?

No. Here's why:

The immune system is constantly processing new threats. Even in a sterile environment, a baby’s immune system deals with thousands of new bacteria and viruses daily from their skin, mouth, and gut.

Vaccines use only a tiny portion of immune system capacity. The current vaccine schedule presents a very small immune challenge compared to what babies naturally encounter. In fact, today’s vaccines are far more refined and use fewer antigens than vaccines from decades ago.

Scientific consensus: Decades of research and monitoring show that giving multiple vaccines in infancy is safe and effective. Serious side effects are rare, and the benefits — preventing severe illness, disability, or death — far outweigh the risks.

If you’re ever unsure or want to space out vaccines for personal reasons, that’s something you can talk to your pediatrician about. They can help you make a plan that balances protection and comfort.

Would you like a breakdown of what each early-life vaccine protects against?

**10-)Why are vaccines given to babies so early? Doesn’t breast milk protect them?**

ChatGPT:

Great question — and it's one many parents ask. Here's a clear breakdown:

1. Why vaccines are given early:

Babies are born with immature immune systems, which makes them especially vulnerable to serious infections. Vaccines are given early to:

Protect during the most vulnerable time: Some diseases (like whooping cough, Haemophilus influenzae type b, and pneumococcal disease) can be especially dangerous — even fatal — in infants under 6 months old.

Build immunity before exposure: It takes time for the immune system to respond to vaccines and build protective antibodies, so starting early means they’re protected sooner.

Prevent outbreaks: Diseases like measles and pertussis spread quickly. Vaccinating on schedule helps prevent community-wide outbreaks, especially in places where herd immunity is important.

2. What about breast milk protection?

Yes — breast milk does provide some immune protection, especially:

Antibodies (mainly IgA) from the mother that help protect against certain gut and respiratory infections.

Immune cells and enzymes that can support immune development.

BUT:

The protection is limited and temporary.

Breast milk doesn’t protect well against many of the specific, dangerous diseases that vaccines target (like polio, tetanus, or Hib).

Passive immunity from the mother fades quickly in the first few months.

Summary:

Vaccines are early because disease risk is early.

Breast milk helps, but can’t replace vaccines.

The two actually work together: breast milk supports the immune system, and vaccines train it to fight specific threats.

Let me know if you want a breakdown of a specific vaccine or disease.

**11-)Are mRNA vaccines safe?**

ChatGPT:

Yes, mRNA vaccines are generally safe for the vast majority of people. They have been extensively tested in clinical trials and closely monitored since their rollout during the COVID-19 pandemic. Here's a breakdown:

✅ What we know about mRNA vaccine safety

Thorough Testing:

mRNA vaccines like Pfizer-BioNTech and Moderna went through rigorous clinical trials involving tens of thousands of participants before approval.

Monitoring Millions:

Since 2020, billions of doses have been administered worldwide. This has provided real-world data on rare side effects and long-term outcomes.

Common Side Effects:

Most people experience mild, short-term reactions:

Sore arm

Fatigue

Headache

Fever or chills

These typically resolve in 1–3 days.

Rare Side Effects:

Myocarditis (heart inflammation): Rarely seen, especially in younger males after the second dose. Most cases are mild and recover without complications.

Allergic reactions: Anaphylaxis is extremely rare and treatable.

No evidence of fertility issues or DNA alteration.

No Live Virus:

mRNA vaccines don’t contain the virus, can’t cause COVID-19, and don’t change your genetic code.

📚 What leading health agencies say:

CDC, WHO, EMA, and other major health organizations continue to recommend mRNA vaccines due to their favorable safety profile.

Benefits (e.g., protection from severe illness and hospitalization) far outweigh the risks for most people.

🔍 Still unsure?

If you have a specific condition (e.g., autoimmune disease, pregnancy, history of allergic reactions), talk to a healthcare provider. They can weigh individual risks and benefits with you.

Would you like info on a particular mRNA vaccine (Pfizer, Moderna, etc.) or group (kids, pregnant people, immunocompromised)?

**12-)Can vaccines cause long-term health problems?**

ChatGPT:

Vaccines are extensively studied for safety before and after approval, and the overwhelming scientific consensus is that vaccines do not cause long-term health problems for the vast majority of people.

Here's what we know:

✅ Extensive Safety Monitoring

Vaccines go through years of clinical trials before approval.

After approval, they are continuously monitored by agencies like the CDC, FDA, and WHO for rare or long-term effects.

⚠️ Possible Rare Side Effects

Like any medical intervention, vaccines can have side effects, most of which are mild (e.g., soreness, fever).

Serious side effects are very rare (e.g., severe allergic reactions or myocarditis after some COVID-19 vaccines), and health authorities actively track and investigate these.

Long-term effects from vaccines (appearing months or years later) are extremely rare, and no credible evidence links vaccines to chronic conditions like autism, autoimmune diseases, or infertility.

🧬 How Vaccines Work Supports Their Safety

Vaccines generally don’t stay in the body long-term.

They train your immune system, then are cleared out—unlike infections, which can cause lingering damage.

What About COVID-19 Vaccines?

COVID-19 vaccines were developed rapidly, but not rushed: they followed all standard safety steps.

Monitoring has shown they are safe and effective, and serious long-term effects remain very rare.

Notably, COVID-19 illness itself is more likely to cause long-term complications ("long COVID") than the vaccine.

Bottom Line:

Vaccines are one of the safest and most effective public health tools we have. While rare side effects can occur, the risk of serious long-term problems is extremely low—and the benefits far outweigh the risks for most people.

If you have concerns about a specific vaccine or condition, talking with a trusted healthcare provider is always a good idea.

**13-)Are flu vaccines necessary? Why do we need to get flu vaccines every year?**

ChatGPT:

Flu vaccines are important, especially for protecting vulnerable populations, and yes, we need to get them every year for several key reasons:

🦠 Why Are Flu Vaccines Necessary?

Prevent illness and complications: The flu can be serious—even deadly—for older adults, young children, pregnant women, and people with certain medical conditions (e.g., asthma, diabetes, heart disease).

Reduce spread: Getting vaccinated helps prevent you from spreading the virus to others, including those who can't get vaccinated or who may not respond well to the vaccine.

Lower hospitalizations and deaths: Annual flu vaccination prevents millions of illnesses and tens of thousands of hospitalizations and deaths each year, according to the CDC and WHO.

🔄 Why Do We Need Flu Vaccines Every Year?

The virus mutates quickly: The flu virus is constantly evolving. There are many different strains (like H1N1, H3N2, etc.), and they can change from year to year. These small changes (called antigenic drift) make last year's vaccine less effective.

Updated vaccines: Each year, scientists track circulating flu strains and develop a new vaccine that targets the strains expected to be most common that season.

Waning immunity: Even if the strains didn’t change, the immunity you get from a flu shot doesn’t last forever. It declines over time—especially after 6–8 months.

🧬 Is It 100% Effective?

No. The flu vaccine isn’t perfect, but even if you do catch the flu, it can reduce the severity and duration of illness and the risk of complications.

✅ Bottom Line

Yes, flu vaccines are necessary—especially annually—because the virus changes and your immunity fades. They're a safe and effective way to reduce personal risk and protect public health.

Let me know if you want help deciding whether you personally should get one or have specific health conditions to consider.

**14-)Is it true that big pharmaceutical companies knowingly endanger children’s health by producing and promoting vaccines?**

ChatGPT:

No, it is not true that big pharmaceutical companies knowingly endanger children’s health by producing and promoting vaccines. This claim is a common element of vaccine misinformation and conspiracy theories, but it does not reflect the evidence-based consensus of the scientific and medical communities.

Here are the key facts:

✅ 1. Vaccines Are Rigorously Tested for Safety

Vaccines undergo multiple phases of clinical trials involving tens of thousands of participants, including children where appropriate.

These trials are reviewed by independent experts and regulatory agencies like the U.S. FDA, European Medicines Agency (EMA), and the World Health Organization (WHO).

Even after approval, vaccines are subject to ongoing safety monitoring through systems like VAERS (U.S.), EudraVigilance (EU), and others globally.

✅ 2. Vaccines Have Saved Millions of Lives

Childhood vaccines have nearly eradicated or dramatically reduced diseases like measles, polio, diphtheria, and rubella.

The World Health Organization estimates that vaccines save 4–5 million lives per year.

✅ 3. Pharmaceutical Companies Are Heavily Regulated

Drug and vaccine manufacturers operate under strict legal and ethical guidelines.

Regulatory bodies can and do halt production or fine companies for violations. Whistleblower protections also exist to expose any misconduct.

✅ 4. No Evidence of a Conspiracy to Harm Children

Allegations that pharma companies deliberately harm children are not supported by credible evidence.

While pharmaceutical companies are profit-driven and have faced legitimate criticisms (e.g., price gouging, opioid marketing), that is distinct from the false claim that they intentionally produce harmful vaccines.

🧠 Critical Thinking Tip:

It’s important to differentiate between healthy skepticism (e.g., asking how drugs are approved or priced) and conspiratorial thinking, which often relies on anecdote, misinformation, or mistrust of all institutions.

If you’re concerned about vaccine safety, consult sources like:

CDC (Centers for Disease Control and Prevention)

WHO

Your healthcare provider or pediatrician

They can provide up-to-date, science-based guidance.

Would you like help evaluating a specific claim or article about vaccines?

**15-)HPV is the main cause of cervical cancer. Then, should boys also get the HPV vaccine?**

ChatGPT:

Yes, boys should also get the HPV vaccine—and here's why:

1. HPV affects all genders

HPV (human papillomavirus) is not just a cause of cervical cancer. It can also lead to:

Penile cancer

Anal cancer

Oropharyngeal (throat, tongue, and tonsil) cancers

Genital warts (common in both males and females)

2. Vaccinating boys helps stop the spread

HPV is transmitted through intimate skin-to-skin contact, including sexual activity.

Vaccinating boys reduces the overall amount of HPV circulating in the population, which protects everyone—including their future partners.

3. It’s most effective when given early

The HPV vaccine works best when given before any exposure to the virus, typically recommended around ages 11–12, but it can be given as early as age 9.

4. HPV-related cancers are rising in men

Oropharyngeal cancer, mostly caused by HPV, is now more common in men than cervical cancer is in women in some countries like the U.S.

In Summary:

The HPV vaccine is not just for girls. It's a powerful cancer-prevention tool for everyone. Boys should definitely be vaccinated to protect their own health and contribute to public health.

Let me know if you'd like recommendations or schedules for HPV vaccination.

**16-)Can people with egg allergies get vaccinated?**

ChatGPT:

Yes, most people with egg allergies can safely receive vaccines, including those that are made using egg-based manufacturing processes. However, it depends on the specific vaccine and the severity of the allergy. Here's a breakdown:

🥚 Egg Allergy & Vaccines: Key Points

✅ Safe for Most People With Egg Allergy:

Flu vaccines (Influenza)

Most flu vaccines are made using egg-based methods.

Current guidelines (CDC, WHO, etc.) say they are safe even for people with egg allergies, including those with severe reactions.

Observation for 15–30 minutes after vaccination may be recommended in some cases.

MMR (Measles, Mumps, Rubella) vaccine

Grown in chick embryo fibroblasts, not egg protein, so it's safe for people with egg allergies.

COVID-19 vaccines

mRNA vaccines like Pfizer-BioNTech and Moderna do not contain egg proteins.

Safe for people with egg allergies.

❗Caution or Alternatives May Be Needed:

Yellow fever vaccine

This does contain egg protein, and may cause reactions in egg-allergic individuals.

People with severe egg allergies should consult an allergist before receiving this vaccine.

Sometimes it's given under medical supervision with precautions (like desensitization or graded dosing).

🩺 What You Should Do:

Tell your healthcare provider about your allergy before getting vaccinated.

Ask about the specific vaccine ingredients if you're unsure.

Get vaccinated in a setting equipped to handle allergic reactions if you have a history of severe anaphylaxis.

If you want to know about a specific vaccine (like yellow fever or rabies), I can give more details.

**17-)Do COVID-19 vaccines cause heart disease?**

ChatGPT:

No, COVID-19 vaccines do not cause heart disease in the general population. However, there are some important nuances to be aware of:

🧬 What’s the Evidence?

Extensive studies show that COVID-19 vaccines are safe and effective for the vast majority of people.

However, rare cases of myocarditis and pericarditis (inflammation of the heart or the lining around it) have been reported, particularly:

In younger males (usually under 30)

More often after mRNA vaccines (Pfizer-BioNTech and Moderna)

Typically within a few days after the second dose

💡 Key Points:

These cases are rare and usually mild, with most people recovering quickly.

Risk of heart complications from COVID-19 infection itself is significantly higher than from the vaccine.

Large-scale monitoring by health agencies (CDC, EMA, WHO) continues to show the benefits of vaccination far outweigh the risks.

🩺 Should You Be Concerned?

Unless you have a specific medical condition or have been advised by your doctor, there’s no reason to avoid COVID-19 vaccination due to concerns about heart disease. If you have a personal or family history of cardiac issues, talk to your healthcare provider for tailored advice.

Would you like help finding official data or studies on this topic?

**18-)If diseases like Hepatitis B still exist despite vaccination, doesn’t that mean the vaccine isn’t effective?**

ChatGPT:

That's a really common and reasonable question. The short answer is: Hepatitis B vaccines are highly effective, but no vaccine is 100% perfect, and several factors explain why the disease still exists despite widespread vaccination efforts.

Here's a breakdown:

✅ Effectiveness of the Hepatitis B Vaccine

The Hepatitis B vaccine is over 95% effective at preventing infection in people who complete the full series (usually 3 doses).

It provides long-lasting protection, often for life, even if antibody levels wane over time.

So why does Hepatitis B still exist?

1. Not everyone is vaccinated

In many parts of the world, especially lower-income countries, vaccine coverage is incomplete.

Some people miss vaccination as infants (when it's most protective), or don’t complete the series.

2. Chronic infections from before vaccines existed

Hepatitis B can become a lifelong, chronic infection if acquired at birth or in early childhood.

Many people alive today were infected before vaccines were widely available (introduced in the 1980s and 1990s).

These chronic carriers can still transmit the virus.

3. Perinatal transmission

If a baby is born to a mother with Hepatitis B and doesn't get timely birth-dose vaccination and possibly hepatitis B immunoglobulin, the risk of transmission is high.

4. Immunocompromised people

Some people (e.g., those with HIV, on chemotherapy, or with kidney disease) may not respond fully to the vaccine and remain at risk despite being vaccinated.

Bottom line:

The existence of Hepatitis B today doesn't mean the vaccine doesn't work—it means we still need better global coverage, early vaccination, and screening and treatment for those already infected.

Let me know if you’d like a visual breakdown or the stats on global Hepatitis B burden vs. vaccine rollout.

**19-)Do vaccines cause infertility?**

ChatGPT:

No, vaccines do not cause infertility.

This concern has been studied extensively, particularly with vaccines like the COVID-19 vaccines, HPV vaccine, and others. Here's a breakdown of what the scientific evidence shows:

✅ What the research says:

No evidence links any authorized vaccine to infertility in either men or women.

COVID-19 vaccines, in particular, have been studied in depth. Multiple large studies and real-world data show no impact on fertility, pregnancy outcomes, or future reproductive health.

Some misinformation arose due to misunderstanding of how vaccines work or unverified claims on social media, but these have been debunked by medical experts and public health organizations.

🧠 How vaccines work:

Vaccines train your immune system to recognize and fight specific pathogens. They do not interact with reproductive organs or hormones in any way that could cause infertility.

📚 Trusted sources:

Centers for Disease Control and Prevention (CDC)

World Health Organization (WHO)

American College of Obstetricians and Gynecologists (ACOG)

Peer-reviewed studies from journals like JAMA and The Lancet

If you have personal concerns about fertility or vaccines, it’s always best to talk with a healthcare provider who can address your individual health situation.

**20-) Can I get sick from the viruses or bacteria in a vaccine?**

ChatGPT:

In most cases, no, you cannot get sick from the viruses or bacteria in a vaccine—especially not in the way the disease itself would make you sick. Here’s why:

🔬 Types of Vaccines and Why They’re Safe

Inactivated (killed) vaccines

Example: Polio (IPV), Hepatitis A

✅ These contain viruses or bacteria that have been killed. They cannot cause disease.

Live attenuated vaccines

Example: MMR (measles, mumps, rubella), Varicella (chickenpox)

⚠️ These use a weakened form of the virus that replicates poorly.

✅ Healthy people almost never get sick from them.

⚠️ People with weakened immune systems may need to avoid them.

Subunit, recombinant, conjugate, or mRNA vaccines

Example: COVID-19 (mRNA), HPV, Hib, Hepatitis B

✅ These do not contain any live virus or bacteria—just a piece of it (like a protein). You cannot get the disease from these.

🧠 But what about side effects?

Mild symptoms, like a fever or sore arm, can happen. That’s your immune system responding—it means the vaccine is working.

These are not the disease itself and are usually short-lived.

🛑 Can vaccines ever cause illness?

Extremely rarely, live vaccines may cause mild versions of the disease in people with very weak immune systems (like those undergoing chemotherapy or with certain immune conditions).

This is why doctors screen people before giving those types of vaccines.

If you have a specific vaccine in mind or a health condition you’re concerned about, I can help explain that one in more detail.
